# State-level prescription drug monitoring program mandates and adolescent injection drug use in the United States, 1995–2017: A difference-in-differences analysis

**DOI:** 10.1371/journal.pmed.1003272

**Published:** 2020-09-25

**Authors:** Joel J. Earlywine, Scott E. Hadland, Julia Raifman

**Affiliations:** 1 Department of Health Law, Policy, and Management, Boston University School of Public Health, Boston, Massachusetts, United States of America; 2 Department of Health Services, University of Washington School of Public Health, Seattle, Washington, United States of America; 3 Grayken Center for Addiction/Department of Pediatrics, Boston Medical Center, Boston, Massachusetts, United States of America; 4 Division of General Pediatrics, Department of Pediatrics, Boston University School of Medicine, Boston, Massachusetts, United States of America; Maryland Treatment Centers, UNITED STATES

## Abstract

**Background:**

Prescription opioid misuse is an ongoing crisis and a risk factor for injection drug use (IDU). Few studies have evaluated strategies for preventing opioid or IDU initiation among adolescents. We evaluated changes in the proportion of adolescents reporting IDU before and after prescription drug monitoring program (PDMP) mandates were implemented in 18 states compared to 29 states without such mandates.

**Methods and findings:**

This difference-in-differences analysis used biannual Youth Risk Behavioral Surveillance System (YRBSS) data representative of adolescents 17 to 18 years old across 47 states from 1995 to 2017. We compared changes in adolescent IDU in 18 states with and 29 states without PDMP mandates. Among 331,025 adolescents, 51.7% identified as male, 62.1% as non-Hispanic white, 17.4% as non-Hispanic black, and 14.6% as Hispanic. Overall, 3.5% reported IDU during the 2 years prior to PDMP mandates. In the final multivariable difference-in-differences model, we included individual age, sex, and race/ethnicity, as well as state and year as covariates from the YRBSS. We also included state- and year-specific poverty rates based on US Census Bureau data. Additionally, we controlled for state implementation of (non-mandated) PDMPs before states subsequently implemented mandates and pill mill laws. We conducted several sensitivity analyses, including repeating our main analysis using a logistic, rather than linear, model, and with a lead indicator on PDMP mandate implementation, a lag indicator, and alternative policy implementation dates. PDMP mandates were associated with a 1.5 percentage point reduction (95% CI −2.3 to −0.6 percentage points; *p =* 0.001) in adolescent IDU, on average over the years following mandate implementation, a relative reduction of 42.9% (95% CI −65.7% to −17.1%). The association of PDMP mandates with this reduction persisted at least 4 years beyond implementation. Sensitivity analyses were consistent with the main results. Limitations include the multi-stepped causal pathway from PDMP mandate implementation to changes in IDU and the potential for omitted state-level time-varying confounders.

**Conclusions:**

Our analysis indicated that PDMP mandates were associated with a reduction in adolescent IDU, providing empirical evidence that such mandates may prevent adolescents from initiating IDU. Policymakers might consider PDMP mandates as a potential strategy for preventing adolescent IDU.

## Abstract 2

Joel Earlywine and colleagues report on adolescent injection drug use and state prescription drug monitoring mandates in the United States.

## Author summary

**Why was this study done?:**

- The misuse of prescription opioids is a critical step in the trajectory toward injection of opioids and other substances. Intervening early on in this trajectory may prevent transitions to injection drug use and related health harms.
- Prescription drug monitoring programs track prescriptions of controlled substances, including opioids, and are designed to limit excessive prescriptions of opioids, but many states do not require clinicians to use this tool. As of January 1, 2017, 18 states in our sample had implemented laws that require the use of a state prescription drug monitoring program.
- We evaluated whether state prescription drug monitoring program mandates were associated with changes in adolescent injection drug use.

**What did the researchers do and find?:**

- Using data from over 330,000 US high school students 17–18 years old, we compared changes in self-reported injection drug use over time in states that implemented prescription drug monitoring program mandates versus states that did not.
- Our results show that injection drug use among 17 and 18 year olds declined after states implemented prescription drug monitoring program mandates compared to states that did not.

**What do these findings mean?:**

- The reduction in adolescent injection drug use associated with prescription drug monitoring program mandates is equivalent to an estimated 81,754 (95% CI 32,620 to 125,357) fewer adolescents initiating injection drug use over a year if states without prescription drug monitoring program mandates implemented these mandates.
- This study finds that prescription drug monitoring program mandates may be an important and effective strategy to prevent adolescent injection drug use.
- Policymakers might consider implementing prescription drug monitoring program mandates along with other substance use policies such as expanded addiction treatment, naloxone distribution, safe prescribing interventions, and harm reduction services.

## Introduction

Drug overdose deaths in the US have increased more than 4-fold in under 2 decades. Of more than 70,237 overdose deaths in 2017, two-thirds involved opioids [1]. Injection drug use (IDU), particularly of opioids, is a major driver of rising overdose mortality. Between 2004 and 2014, addiction treatment admissions among adolescents 12–17 years old involving any opioid injection increased 103%; heroin and prescription opioid injection increased 97% and 194%, respectively [2]. Increasing prevalence of adolescent IDU has coincided with a 3-fold rise in opioid overdose deaths among adolescents since 1999 [3], as well as recent outbreaks of human immunodeficiency virus (HIV) and hepatitis C virus (HCV) among young people [4,5]. IDU is associated with increased risk of overdose [6] and acquisition of HIV, HCV, and other infections [7,8].

Prescription opioid misuse is commonly a critical step in the trajectory towards injection of opioids and other substances [9–13]. In nationally representative studies, adolescents who misused prescription opioids were 13 to 17 times more likely to transition to heroin [11,13] and 14 times more likely to transition to IDU [13] compared to those who did not. Policies that may intervene early on in this trajectory deserve further scrutiny.

The majority of adolescents who misuse prescription opioids obtain them from another person [14]; therefore, reducing the oversupply of prescription opioids in the general population that could be diverted to family members, friends, or illicit markets may be an important strategy for preventing adolescent opioid misuse and the transition to IDU [15]. To limit excessive opioid prescriptions, 49 US states have implemented statewide prescription drug monitoring programs (PDMPs). PDMPs are repositories of recently dispensed prescriptions for controlled substances, and help prescribers limit excessive prescriptions to patients receiving opioids from multiple prescribers. Implementation of PDMPs is associated with a subsequent reduction in prescriptions dispensed [16,17], decreases in drug diversion [18], and reduced opioid misuse among adults [19,20].

Some states have implemented laws mandating use of PDMPs by all opioid prescribers [21]. Such “PDMP mandates” are associated with reductions in opioid-related overdose rates [22–24], but have also been critiqued because reducing the opioid supply may drive individuals with opioid use disorder to transition to heroin and fentanyl [23,25,26]. It is possible that PDMP mandates may affect adolescents differently from adults by preventing the initiation or progression of opioid misuse, a common early step in the trajectory towards drug injection among adolescents. Investigating whether PDMP mandates prevent young people from initiating drug use could contribute to policymaker decisions about PDMP mandates [27].

We aimed to evaluate whether PDMP mandates were associated with self-reported IDU among adolescents using 1995 to 2017 Youth Risk Behavioral Surveillance Survey (YRBSS) data representative of adolescents in 47 US states (the YRBSS only added a question on prescription opioid misuse in 2017, preventing a longitudinal analysis with this outcome). We evaluated the relationship between PDMP mandates and adolescent IDU through a difference-in-differences analysis. We hypothesized that states that implemented PDMP mandates would experience subsequent reductions in adolescent IDU relative to states without such mandates.

## Methods

### Sample

We used YRBSS data on adolescent demographic characteristics and health risk behaviors. The Centers for Disease Control and Prevention (CDC) partners with state health and education departments to collect YRBSS data through 2-stage sampling of high schools and classrooms [28]. The CDC only releases data for states with greater than 60% participation. We used all years of available data for all 47 participating states (January 1, 1995, to December 31, 2017) [28]. We decided a priori to restrict the sample to adolescents aged 17–18 years to focus on those most likely to report IDU, and to increase power to detect potential effects of PDMP mandates [11,29]. We included all students with complete data on IDU, age, sex, and race/ethnicity.

We used only de-identified data for this study. The Boston University Medical Campus Institutional Review Board approved exemption from human participants review for this study. Although we did not formally file a pre-analysis plan, the exposure, outcomes, and analyses were based on ex ante hypotheses, with the exception of the event study analysis, which was added to further test the main model assumptions. This study followed STROBE reporting guidelines (see S1 STROBE Checklist).

### Variables

The main exposure of interest was implementation of state laws that mandate use of PDMPs by prescribers (i.e., “PDMP mandates”). We identified 18 states with and 29 states without a PDMP mandate in place on or before January 1, 2017. Due to differences in mandate implementation dates from different data sources that may contribute to varying results [30], we used data from the Prescription Drug Monitoring Program Training and Technical Assistance Center [31] and the National Alliance for Model State Drug Laws [32] to determine mandate implementation dates. We compared dates from both sources and, where we found discrepancies, read legislation and contacted states’ PDMP administrators to determine the implementation date [23]. We list such discrepancies and how they were resolved in S1 Table.

The main outcome of interest was any (i.e., lifetime) history of IDU among adolescents, based on students’ responses to the question, “During your life, how many times have you used a needle to inject any illegal drug into your body?” Responses consisted of “never,” “one time,” and “two or more times.” Adolescent IDU was defined as ever having injected, i.e., on at least 1 occasion.

We included individual age, sex, and race/ethnicity, as well as state and year as covariates from the YRBSS. We also included state- and year-specific poverty rate as a covariate based on US Census Bureau data. Additionally, we controlled for state implementation of (non-mandated) PDMPs before states subsequently implemented mandates and pill mill laws [33,34].

### Statistical analysis

We evaluated the relationship between state PDMP mandates and adolescent IDU using a difference-in-differences design [35,36]. This approach compared changes in adolescent IDU before and after states implemented PDMP mandates relative to changes in adolescent IDU over time in states without a PDMP mandate. Difference-in-differences analyses are a rigorous form of policy analysis with a comparison group that approximates the counterfactual outcomes that would have occurred in the absence of policy changes [37,38].

Difference-in-differences analyses in this case require that baseline temporal trends in adolescent IDU be equivalent until the year of the law implementation in states that implemented a PDMP mandate relative to comparison states that did not implement mandates. Common trends between the 2 groups before mandates were implemented suggest that continued parallel trends could have been expected in the absence of any policy change and indicate that the comparison group provides an appropriate approximation of the counterfactual [39]. We tested this assumption by repeating the main analytical model for the period prior to PDMP mandate implementation in a linear regression analysis of adolescent IDU, with the main exposure of interest being an interaction term for a linear year trend and a binary indicator for whether states went on to implement mandates. This model also adjusted for differing temporal trends in each state, by interacting state and year, and controlled for age, sex, race/ethnicity, state, year, poverty rate, a binary indicator for living in a state with a non-mandated PDMP after implementation, as well as a binary indicator for living in a state after implementation of a pill mill law.

In our main difference-in-differences analysis, the exposure of interest was a binary indicator for living in a PDMP mandate state after the mandate was implemented. We adjusted for state using state fixed effects, which controlled for all time-invariant state characteristics. By using state fixed effects, we focused on relative changes in IDU within each state, rather than comparing levels of IDU between different states. We also included year fixed effects to control for any exogenous shocks to adolescent IDU common to all states during a given year. We included a state-specific linear time trend to adjust for temporal trends in IDU in each state. As time-varying confounders, we included a binary indicator for living in a state after implementation of any non-mandated PDMP, as well as a binary indicator for living in a state after implementation of a pill mill law. We also adjusted for age, sex, race/ethnicity, and poverty rate. We used a linear regression model because maximum likelihood models can underestimate standard errors in the presence of fixed effects [40]. We used Taylor-series linearized standard errors clustered at the state level to account for serial correlation and the state-level intervention [41]. Finally, using US Census Bureau data on adolescents 17 to 18 years of age in 2017, we estimated the number of adolescents for whom initiation of IDU would have been averted if states without PDMP mandates had implemented PDMP mandates.

To additionally test the equivalent trends assumption and to assess the association of PDMP mandates over time, we fit an event study model [42]. The event study model replaced the main exposure with binary variables of leads and lags of PDMP mandates from 11 years before mandates were implemented to 4 to 5 years after. Adolescents in states that did not implement PDMP mandates were coded as 0 for all of these binary variables. This approach further tests for differences in IDU between states with PDMP mandates and states without PDMP mandates before the mandates were implemented. After PDMP mandates were implemented, the event study model can capture heterogeneous treatment effects by year after implementation and shows whether the association persisted over time. To supplement the event study estimates, we plotted the unadjusted prevalence of adolescent IDU over time relative to PDMP mandate implementation. Since non-mandated states do not experience a mandate, we assigned a placebo mandate year to non-mandated states equivalent to the median mandate year (2013) among PDMP mandate states (S1 Fig) [43].

We conducted several sensitivity analyses. First, we repeated our main analysis in a logistic, rather than linear, regression model. Second, we repeated our main analysis with a lead on the PDMP mandate implementation indicator, i.e., a binary variable that captured that a state would go on to implement a PDMP mandate 1 to 2 years in the future. This analysis would detect whether adolescent IDU had already begun to decline prior to PDMP mandate implementation in states with PDMP mandates. Third, we tested a lagged PDMP mandate, i.e., a binary indicator for having implemented a PDMP mandate 1 to 2 years prior, which allowed us to assess whether the associations of PDMP mandates with adolescent IDU persisted. Fourth, because states implemented non-mandated PDMPs before implementing mandate use laws, we restricted the sample to after states implemented non-mandated PDMPs to assess whether results were sensitive to the exclusion of the original non-mandated PDMP. Lastly, we repeated our main analysis using alternative mandate dates when data sources did not agree. Analyses were conducted using Stata, version 15.1 (StataCorp). All statistical tests were 2-sided and considered significant at *p* < 0.05.

## Results

From 1995 to 2017, there were 331,025 students 17 to 18 years of age who participated in the YRBSS and had complete data for IDU, age, sex, and race/ethnicity. Among these students, 51.7% identified as male, 62.1% as non-Hispanic white, 17.4% as non-Hispanic black, and 14.6% as Hispanic (Table 1). Overall, 2.7% (95% CI 2.5% to 2.8%) of students reported lifetime IDU. Among males, 3.6% (95% CI 3.4% to 3.9%) reported lifetime IDU, compared to 1.6% (95% CI 1.4% to 1.8%) of females. Among students identifying as non-Hispanic white, 2.2% (95% CI 2.0% to 2.5%) reported lifetime IDU; non-Hispanic black, 3.0% (95% CI 2.6% to 3.4%); Hispanic, 3.5% (95% CI 2.8% to 4.2%); and other race/ethnicity, 4.0% (95% CI 3.0% to 5.0%). From 1995 to 2017, the prevalence of lifetime IDU increased from 2.2% (95% CI 1.8% to 2.6%) to 3.0% (95% CI 2.2% to 3.9%).

**Table 1:** Participant characteristics and injection drug use (1995–2017).

Characteristic	Number (%) of students 17 and 18 years old (*n* = 331,025)^a^	Number (%) of students reporting injection drug use (*n* = 9,948)
Sex		
Female	162,706 (48.3)	3,085 (1.6)
Male	168,319 (51.7)	6,863 (3.6)
Age		
17 years old	223,518 (62.3)	5,893 (2.3)
18 years old	107,507 (37.7)	4,055 (3.3)
Race/ethnicity		
White	196,154 (62.1)	4,617 (2.2)
Black or African American	50,674 (17.4)	1,692 (3.0)
Hispanic/Latinx	48,659 (14.6)	2,154 (3.5)
Other race/ethnicity	35,538 (5.9)	1,485 (4.0)
Poverty^b^	— (19.8)	—

^a^All numbers are unweighted while percentages are weighted to be representative of the state population of high school students by student sex, age, and race/ethnicity. Sex, age, and race/ethnicity are based on participant self-report.

^b^Based on US Census Bureau data aggregated at the state level; thus, the poverty level for adolescents who report injection drug use cannot be reported.

Adolescents in states with and without PDMP mandates were similar with respect to sex, age, and race/ethnicity (Table 2). There were statistically significant differences in poverty rates between PDMP mandate states and comparison states.

**Table 2:** Participant characteristics in mandated versus non-mandated states among 17 and 18 year olds across all years of the YRBSS (1995–2017).

Characteristic	Number (%)^a^		*p*-Value
	Mandated states^b^ (*n* = 123,985)	Non-mandated states^c^ (*n* = 207,040)	
Sex			0.07
Female	61,068 (48.0)	101,638 (48.4)	
Male	62,917 (52.0)	105,402 (51.6)	
Age			0.15
17 years old	84,729 (63.8)	138,789 (61.4)	
18 years old	39,256 (36.2)	68,251 (38.6)	
Race/ethnicity			0.15
White	73,889 (68.9)	122,265 (58.2)	
Black/African American	13,484 (13.5)	37,190 (19.6)	
Hispanic/Latinx	23,561 (11.0)	25,098 (16.7)	
Other race/ethnicity	13,051 (6.5)	22,487 (5.5)	
Poverty	— (18.8)	— (20.5)	<0.001^d^

All *p*-values reported come from chi-squared tests unless otherwise noted.

^a^All numbers are unweighted while percentages are weighted to be representative of the state population of high school students by student sex, age, and race/ethnicity. Sex, age, and race/ethnicity are based on participant self-report.

^b^Eighteen states.

^c^Twenty-nine states.

^d^*p-*Value is from the *t* test.

YRBSS, Youth Risk Behavioral Surveillance System.

**Fig 1:**
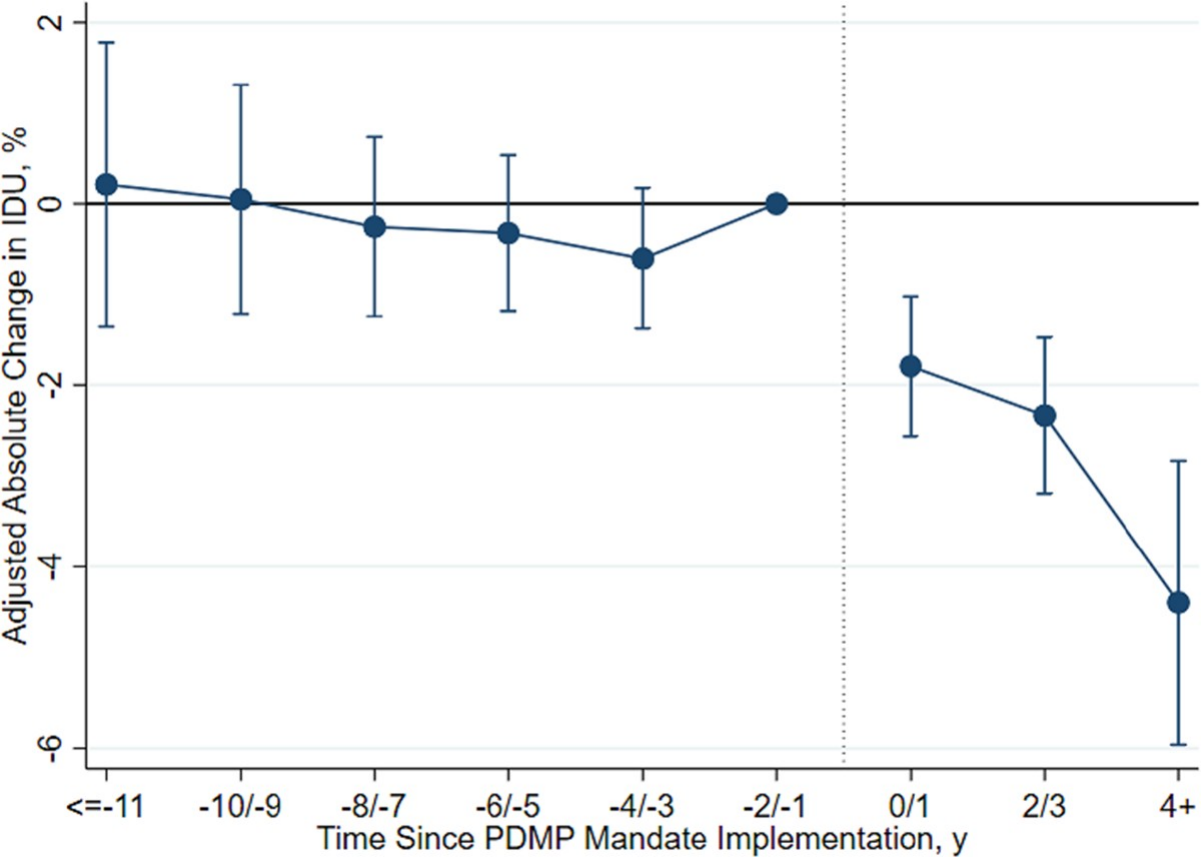
Event study estimates for the association of PDMP mandate implementation with adolescent IDU relative to comparison states. This graph presents event study estimates for the association of PDMP mandate implementation with adolescent IDU relative to comparison states. For this graph, the PDMP mandate implementation variable is a series of binary indicators denoting the year relative to PDMP mandate implementation. Adolescents in comparison states are coded as 0 for this series of binary indicators. The period before PDMP mandate implementation (−2/−1) was the reference period. Each point compares the difference in IDU between states that implemented a PDMP mandate and comparison states. The error bars represent 95% confidence intervals (where period −2/−1 does not have a 95% confidence interval because it was the reference period). IDU, injection drug use; PDMP, prescription drug monitoring program.

Temporal trends in adolescent IDU in PDMP mandate states and comparison states did not differ prior to implementation of PDMP mandates (average difference in IDU prevalence, 0.03 percentage points; 95% CI <−0.01 to 0.06; *p* = 0.054; Figs 1 and S1; S2 Table). The proportion of adolescents reporting IDU was also similar between PDMP mandate and comparison states prior to PDMP mandates [39].

Adolescent IDU decreased in PDMP mandate states following PDMP mandate implementation (Fig 1). In the 1 to 2 years prior to PDMP mandates, 3.5% of adolescents in both PDMP mandate and comparison states reported any IDU (Table 3). PDMP mandates were associated with a 1.5 percentage point absolute reduction (95% CI −2.3 to −0.6 percentage points; *p =* 0.001) in IDU prevalence on average over the years following mandate implementation relative to comparison states (S3 Table; Fig 1), a relative reduction of 42.9% (95% CI −65.7% to −17.1%). With 5,450,298 adolescents 17–18 years old living in states without PDMP mandates in the US in 2017, this associated reduction translates into approximately 81,754 (95% CI 32,620 to 125,357) fewer adolescents injecting drugs per year if states without PDMP mandates had implemented PDMP mandates (Table 3). The association of PDMP mandates with adolescent IDU reduction persisted at least 4 years after implementation (Fig 1).

**Table 3:** Changes in adolescent IDU in states with a PDMP mandate relative to comparison states.

Measure	Adolescents reporting IDU before PDMP mandate	Net change in adolescents reporting IDU following PDMP mandate (95% CI)
Absolute change in prevalence	3.5%	−1.5 percentage points (−2.3 to −0.6)^a^
Relative change in prevalence	—	−42.9% (−65.7% to −17.1%)
Number of adolescents^b^	190,760	−81,754 (−125,357 to −32,620)

^a^*p* = 0.001.

^b^Based on US Census Bureau data for national population of youth ages 17–18 years in 2017 in comparison states, where the net change represents the number of adolescents for whom IDU would have been averted per year if comparison states had implemented PDMP mandates.

IDU, injection drug use; PDMP, prescription drug monitoring program.

Sensitivity analyses were consistent with the main results. In the model employing multivariable logistic (rather than linear) regression, PDMP mandates were associated with a 44% reduction (95% CI 23% to 60%; *p =* 0.001) in odds of initiating IDU relative to comparison states (S4 Table). A lead indicator for states going on to implement PDMP mandates 1 to 2 years in the future was not associated with adolescent IDU, indicating that declines in IDU did not precede PDMP mandate implementation (S5 Table). A lagged indicator for states implementing PDMP mandates 1 to 2 years prior was associated with a 1.5 percentage point absolute reduction (95% CI −2.4 to −0.6 percentage points; *p =* 0.002) in adolescent IDU, indicating that reductions in IDU associated with PDMP mandates persisted in the years following implementation (S6 Table). When restricting the time period to after states implemented non-mandated PDMPs prior to mandating their use, implementation of a PDMP mandate was associated with a 1.5 percentage point absolute reduction (95% CI −2.2 to −0.7; *p <* 0.001) in adolescent IDU (S7 Table). The estimated association between PDMP mandates and adolescent IDU was consistent when we replicated our main difference-in-differences model with alternative mandate dates (−1.4 percentage points; 95% CI −2.1 to −0.7; *p <* 0.001; S8 Table).

## Discussion

Based on representative data from 17 and 18 year olds in 47 states, 1 in 33 adolescents reported ever having injected drugs in 2017, a 36% increase since 1995. In the 1 to 2 years prior to PDMP mandate implementation, 3.5% of adolescents in both PDMP mandate and comparison states reported IDU. We found that state PDMP mandates were associated with a 1.5 percentage point reduction in IDU relative to comparison states, equivalent to a 43% relative reduction. The association between PDMP mandates and adolescent IDU persisted 4 years after mandate implementation.

These findings have important potential policy implications. We find evidence that PDMP mandates may be playing a protective role for adolescents, suggesting that 81,754 transitions to IDU might be prevented annually by implementation of PDMP mandates. As policymakers consider mandates as a response to the overdose crisis, it is important to evaluate their net effects. Our findings add to a growing body of literature evaluating the effects of PDMPs and PDMP mandates. Haffajee [27] recently expressed a continued need for rigorous PDMP evaluation, especially regarding unintended consequences, like substitution of prescription medications with illicit substances. Heroin and fentanyl, which are commonly injected, are playing a growing role in the opioid epidemic [1]. A concern about PDMP mandates has been that restricting prescriptions for opioids may lead to substitution with cheaper, more potent opioids such as heroin or fentanyl, particularly among adults [23,25,26]. However, we find that this may not be the case for adolescents. We show that adolescents may be substantially benefitting from PDMP mandate implementation, and this could translate to important decreases in harms related to IDU such as fatal and nonfatal overdose, HIV infection, and HCV infection.

PDMPs are just one tool in the midst of an evolving and complex drug overdose epidemic. Crises such as these require a multifaceted approach that takes into account the intended and unintended consequences of interventions such as PDMP mandates. This study suggests that PDMP mandates may prevent adolescents from beginning down the path of opioid addiction and IDU, which is important to consider alongside evidence that PDMPs could prompt people who are already using opioids to transition to illicit substances [27]. Enactment of PDMP mandates could complement other evidence-based interventions, such as expanded addiction treatment [44,45], naloxone distribution [46,47], safe prescribing interventions [48], and harm reduction services [49].

Strengths of the study include its use of representative data from nearly all US states and a rigorous difference-in-differences analytical design. The findings were robust in numerous sensitivity analyses, indicating that declines in IDU prevalence among adolescents did not precede PDMP mandate implementation and that the effect of PDMP mandates persisted at least 4 years following implementation; findings were also not dependent on the functional form of the regression. Limitations of this study include a multi-stepped causal pathway from PDMP implementation to changes in IDU. Although 1 study found that PDMPs and PDMP mandate implementation were associated with reductions in treatment admissions involving prescription drug misuse among 12–17 year olds [20], we lacked data to empirically demonstrate that prescription opioid misuse mediated the relationship between PDMP mandates and IDU. Lastly, we cannot rule out the possibility of omitted state-level time-varying confounders.

## Conclusion

We provide evidence that PDMP mandates may be an effective policy to prevent IDU among adolescents. Our study adds to prior evidence that PDMP mandates reduce opioid prescriptions and overdose mortality among adults, and contributes evidence that PDMP mandates may be a useful tool to address the growing concern surrounding substitution to illicit substances. Very few studies have looked at policies that may prevent initiation of IDU. Policymakers might consider PDMP mandates as a strategy to reduce IDU among adolescents.

## Supporting information

S1 STROBE Checklist (DOC)

S1 Fig Unadjusted prevalence of adolescent IDU in PDMP mandate states compared to non-mandate states relative to year of mandate implementation. (DOCX)

S1 Table Mandated PDMP implementation dates and resolutions. (DOCX)

S2 Table Linear analysis of differences in baseline trends in PDMP mandate and non-PDMP-mandate states. (DOCX)

S3 Table Linear difference-in-differences analysis of PDMP mandates: Adolescent IDU in PDMP mandate states relative to non-PDMP-mandate states. (DOCX)

S4 Table Logistic difference-in-differences analysis of PDMP mandates: Adolescent IDU in PDMP mandate states relative to non-PDMP-mandate states. (DOCX)

S5 Table Linear difference-in-differences “lead” analysis of PDMP mandates in the 1–2 years prior to implementation: Adolescent IDU in PDMP mandate states relative to non-PDMP-mandate states. (DOCX)

S6 Table Linear difference-in-differences “lag” analysis of PDMP mandates in the 1–2 years after implementation: Adolescent IDU in PDMP mandate states relative to non-PDMP-mandate states. (DOCX)

S7 Table Linear difference-in-differences analysis of PDMP mandates after non-mandated PDMP implementation: Adolescent IDU in PDMP mandate states relative to non-PDMP-mandate states. (DOCX)

S8 Table Linear difference-in-differences analysis of alternative implementation dates for PDMP mandates: Adolescent IDU in PDMP mandate states relative to non-PDMP-mandate states. (DOCX)

## Abbreviations

HIC: hepatitis C virus
HIV: human immunodeficiency virus
IDU: injection drug use
PDMP: prescription drug monitoring program
YRBSS: Youth Risk Behavioral Surveillance Survey
