# Supplementary material for: State-level prescription drug monitoring program mandates and adolescent injection drug use in the United States, 1995–2017: A difference-in-differences analysis
Source: PLoS Med. 2020 Sep 25;17(9):e1003272. doi: 10.1371/journal.pmed.1003272 (PMC7518580; doi:10.1371/journal.pmed.1003272)
Supplement: S1 Fig — (DOCX) [file pmed.1003272.s002.docx]

**S1 Fig.** Unadjusted Prevalence of Adolescent Injection Drug Use in PDMP Mandate States Compared to Non-Mandate States Relative to Year of Mandate Implementation

**Legend:** This figure shows unadjusted trends in adolescent IDU between mandated and non-mandates states relative to the year of mandate implementation. Sine non-mandated states never experience a PDMP mandate, we assigned a placebo mandate year equivalent to the median year of mandate implementation among PDMP mandate states.
