# Supplementary material for: State-level prescription drug monitoring program mandates and adolescent injection drug use in the United States, 1995–2017: A difference-in-differences analysis
Source: PLoS Med. 2020 Sep 25;17(9):e1003272. doi: 10.1371/journal.pmed.1003272 (PMC7518580; doi:10.1371/journal.pmed.1003272)
Supplement: S1 Table — (DOCX) [file pmed.1003272.s003.docx]

**S1 Table**. Mandated Prescription Drug Monitoring Program Implementation Dates and Resolutions

|  | **Brandeis TTA** | | **NAMSDL** | | **Author Dates** | | **Robustness Checks** | | **Resolutions** |
| --- | --- | --- | --- | --- | --- | --- | --- | --- | --- |
| *US State* | *PDMP Mandate* | *Implementation Date* | *PDMP Mandate* | *Implementation Date* | *PDMP Mandate* | *Implementation Date* | *PDMP Mandate* | *Implementation Date* |  |
| AL | Yes | 3/9/2017 | Not provided |  | No |  | No |  | 540-X-4-.09 Risk and Abuse Mitigation Strategies by Prescribing Physicians |
| AK | Yes | 7/17/2017 | Yes | 7/17/2017 | No |  | No |  | Out of sample |
| AZB | Yes | 7/20/2011 | Yes | 10/16/2017 | No |  | No |  | Out of sample - Section H of A.R.S. § 36-2606 |
| AR | Yes | 7/22/2015 | Yes | 8/1/2017 | No |  | No |  | Out of sample - AR Code § 20-7-604 |
| CA | Yes | 1/1/2017 | Yes | 10/2/2018 | No |  | No |  | Out of sample - Cal.Health & Safety Code § 11165.4 is effective 1/1/2017, however, the CURES database was not ready until 2018, so there was no access until 10/2/2018 |
| CO | Yes | 11/1/2013 | Yes | 5/12/2018 | No |  | No |  | Out of sample - C.R.S.A. § 12-42.5-404(3.6) |
| CT | Yes | 10/1/2015 | Yes | 7/1/2016 | Yes | 10/1/2015 | Yes | 7/1/2016 | Per Public Act 15-198, 10/1/2015 is chosen because of it mandating query of PDMP. Public Act 16-43 only adds and tweaks the original one, but not in a way to affect mandatory query |
| DE | Yes | Not provided | Yes | 1/1/2011 | No |  | Yes | 1/1/2011 | Weak language - must access if provider has reason to believe they are misusing etc. |
| FL | Yes | 7/1/2018 | Yes | 7/1/2018 | No |  | No |  | Out of sample |
| GA | Yes | 7/13/2014 | Yes | 7/1/2018 | No |  | No |  | Out of sample - According to Georgia's PDMP website and house bill 249, effective data of mandatory query is not until 2018 |
| HI | No |  | No |  | No |  | No |  |  |
| ID | No |  | No |  | No |  | No |  |  |
| IL | Yes | 1/1/2018 | Yes | 1/1/2018 | No |  | No |  | Out of sample |
| IN | Yes | 7/1/2014 | Yes | 7/1/2018 | No |  | No |  | Out of sample - Per PDMP administrator: mandatory registration and query fazed from 2019-2021 |
| IA | Yes | 5/14/2017 | Yes | 7/1/2018 | No |  | No |  | Out of sample |
| KS | No |  | No |  | No |  | No |  |  |
| KY | Yes | 7/1/2012 | Yes | 7/20/2012 | Yes | 7/1/2012 | Yes | 7/1/2012 |  |
| LA | Yes | 8/1/2014 | Yes | 6/12/2017 | No |  | Yes | 8/1/2014 | Out of sample - Revised Statutes §40:978 was amended and reenacted in 2017 with Act No. 76 which added the PDMP mandate |
| ME | Yes | 1/1/2017 | Yes | 1/1/2017 | Yes | 1/1/2017 | Yes | 1/1/2017 |  |
| MD | Yes | 7/1/2018 | Yes | 7/1/2018 | No |  | No |  | Out of sample |
| MA | Yes | 1/1/2013 | Yes | 1/1/2013 | Yes | 1/1/2013 | Yes | 1/1/2013 |  |
| MI | Yes | 3/15/2018 | Yes | 6/1/2018 | No |  | No |  | Out of sample |
| MN | Yes | 8/1/2013 | No |  | N/A |  | N/A |  | Does not participate in YRBSS |
| MS | No |  | No |  | No |  | No |  |  |
| MO | No |  | No |  | No |  | No |  |  |
| MT | No |  | No |  | No |  | No |  |  |
| NE | No |  | No |  | No |  | No |  |  |
| NV | Yes | 10/1/2007 | Yes | 10/1/2007 | Yes | 10/1/2007 | Yes | 10/1/2007 |  |
| NH | Yes | 9/1/2016 | No |  | Yes | 9/1/2016 | No |  | RSA 318–B:39 Repealed on 1/1/17 |
| NJ | Yes | 11/1/2015 | Yes | 11/1/2015 | Yes | 11/1/2015 | Yes | 11/1/2015 |  |
| NM | Yes | 8/31/2012 | Yes | 1/1/2017 | Yes | 1/1/2017 | Yes | 1/1/2017 | N. M. S. A. 1978, § 26-1-16.1 |
| NY | Yes | 8/27/2013 | Yes | 8/27/2013 | Yes | 8/27/2013 | Yes | 8/27/2013 |  |
| NC | Yes | 6/29/2017 | Yes | 6/29/2017 | No |  | No |  | Out of sample |
| ND | Yes | 10/1/2014 | Not provided |  | No |  | Yes | 10/1/2014 | 19-03.5-05. Immunity - states nobody is liable to check |
| OH | Yes | 8/10/2011 | Yes | 4/1/2015 | Yes | 4/1/2015 | Yes | 4/1/2015 | House Bill 341 of the 130^th^ Ohio General Assembly mandates use of PDMP |
| OK | Yes | 11/1/2010 | Yes | 11/1/2015 | Yes | 11/1/2015 | Yes | 11/1/2015 | 63 Okl.St.Ann. § 2-309D G. 2. |
| OR | No |  | No |  | N/A |  | N/A |  | Does not participate in YRBSS |
| PA | Yes | 6/30/2015 | Yes | 6/30/2015 | Yes | 6/30/2015 | Yes | 6/30/2015 |  |
| RI | Yes | 1/31/2013 | Yes | 6/28/2016 | Yes | 6/28/2016 | Yes | 6/28/2016 | Gen.Laws 1956, § 21-28-3.32(m) |
| SC | Yes | 5/19/2017 | Yes | 5/19/2017 | No |  | No |  | Out of sample |
| SD | No |  | No |  | No |  | No |  |  |
| TN | Yes | 1/1/2013 | Yes | 4/27/2016 | Yes | 1/1/2013 | Yes | 4/27/2016 | T. C. A. § 53-10-310(e)(1) |
| TX | Yes | 12/25/2016 | Yes | 9/1/2017 | No |  | No |  | Out of sample |
| UT | Yes | 5/10/2016 | Yes | 5/10/2016 | Yes | 5/10/2016 | Yes | 5/10/2016 |  |
| VT | Yes | 11/15/2013 | Yes | 11/15/2013 | Yes | 11/15/2013 | Yes | 11/15/2013 |  |
| VA | Yes | 7/1/2015 | Yes | 7/1/2015 | Yes | 7/1/2015 | Yes | 7/1/2015 |  |
| WA | Yes | 7/1/2013 | Not provided |  | N/A |  | N/A |  | Does not participate in YRBSS |
| WV | Yes | 6/8/2012 | Yes | 6/8/2012 | Yes | 6/8/2012 | Yes | 6/8/2012 |  |
| WI | Yes | 4/1/2017 | Yes | 4/1/2017 | No |  | No |  | Out of sample |
| WY | No |  | No |  | No |  | No |  |  |
