# Supplementary material for: State-level prescription drug monitoring program mandates and adolescent injection drug use in the United States, 1995–2017: A difference-in-differences analysis
Source: PLoS Med. 2020 Sep 25;17(9):e1003272. doi: 10.1371/journal.pmed.1003272 (PMC7518580; doi:10.1371/journal.pmed.1003272)
Supplement: S2 Table — (DOCX) [file pmed.1003272.s004.docx]

**S2 Table.** Linear Analysis of Differences in Baseline Trends in PDMP Mandate and Non-PDMP Mandate States

| **Variables** | **Reported Lifetime Injection Drug Use (N=306,991)** | |
| --- | --- | --- |
|  | Percentage Points | 95% CI |
| PDMP mandate state*year | 0.03 | <-0.01 – 0.06 |
| PDMP (non-mandated) | 0.34 | -0.53 – 1.21 |
| Pill Mill law | 0.45 | -0.07 – 0.97 |
| Sex |  |  |
| Female | *Reference* |  |
| Male | **2.04** | **1.80 – 2.30** |
| Race/Ethnicity |  |  |
| White | *Reference* |  |
| Black/African American | **0.60** | **0.15 – 1.01** |
| Hispanic/Latinx | **1.60** | **0.92 – 2.23** |
| Other race/ethnicity | **2.20** | **1.30 – 3.08** |
| Age |  |  |
| 17 years of age | *Reference* |  |
| 18 years or older | **0.90** | **0.52 – 1.19** |
| Poverty | 0.08 | -0.05 – 0.21 |

Note: Linear probability models include controls for state fixed effects, year fixed effects, and state-specific time trends. Standard errors were clustered by state. Significant (p<0.05) estimates and 95% CIs are bolded.
