# Supplementary material for: State-level prescription drug monitoring program mandates and adolescent injection drug use in the United States, 1995–2017: A difference-in-differences analysis
Source: PLoS Med. 2020 Sep 25;17(9):e1003272. doi: 10.1371/journal.pmed.1003272 (PMC7518580; doi:10.1371/journal.pmed.1003272)
Supplement: S4 Table — (DOCX) [file pmed.1003272.s006.docx]

**S4 Table.** Logistic Difference-in-Differences Analysis of PDMP Mandates: Adolescent Injection Drug Use in PDMP Mandate States Relative to Non-PDMP Mandate States

| **Variables** | **Reported Lifetime Injection Drug Use (N=331,025)** | |
| --- | --- | --- |
|  | Odds Ratio | 95% CI |
| PDMP Mandate Implemented | **0.56** | **0.40 – 0.77** |
| PDMP (non-mandated) | 1.12 | 0.82 – 1.52 |
| Pill Mill law | 1.18 | 0.91 – 1.53 |
| Sex |  |  |
| Female | *Reference* |  |
| Male | **2.33** | **2.06 – 2.62** |
| Race/Ethnicity |  |  |
| White | *Reference* |  |
| Black/African American | **1.31** | **1.10 – 1.55** |
| Hispanic/Latinx | **1.80** | **1.51 – 2.15** |
| Other race/ethnicity | **2.02** | **1.67 – 2.44** |
| Age |  |  |
| 17 years of age | *Reference* |  |
| 18 years or older | **1.40** | **1.24 – 1.57** |
| Poverty | 1.01 | 0.96 – 1.05 |

Note: Logistic regression models include controls for state fixed effects, year fixed effects, and state specific time trends. Standard errors were clustered by state. Significant (p<0.05) estimates and 95% CIs are bolded.
