# Supplementary material for: State-level prescription drug monitoring program mandates and adolescent injection drug use in the United States, 1995–2017: A difference-in-differences analysis
Source: PLoS Med. 2020 Sep 25;17(9):e1003272. doi: 10.1371/journal.pmed.1003272 (PMC7518580; doi:10.1371/journal.pmed.1003272)
Supplement: S5 Table — (DOCX) [file pmed.1003272.s007.docx]

**S5 Table.** Robustness Check Linear Difference-in-Differences "Lead" Analysis of PDMP Mandates in the 1-2 Years Prior: Adolescent Injection Drug Use in PDMP Mandate States Relative to Non-PDMP Mandate States

| **Variables** | **Reported Lifetime Injection Drug Use (N=331,025)** | |
| --- | --- | --- |
|  | Percentage Points | 95% CI |
| Lead: PDMP Mandate Implemented | 0.60 | -0.05 – 1.25 |
| PDMP (non-mandated) | 0.54 | -0.33 – 1.41 |
| Pill Mill law | 0.40 | -0.52 – 1.31 |
| Sex |  |  |
| Female | *Reference* |  |
| Male | **2.00** | **1.80 – 2.30** |
| Race/Ethnicity |  |  |
| White | *Reference* |  |
| Black/African American | **0.70** | **0.30 – 1.00** |
| Hispanic/Latinx | **1.60** | **1.20 – 2.00** |
| Other race/ethnicity | **2.00** | **1.50 – 2.60** |
| Age |  |  |
| 17 years of age | *Reference* |  |
| 18 years or older | **0.90** | **0.70 – 1.10** |
| Poverty | <-0.10 | -0.20 – 0.10 |

Note: Linear probability models include controls for state fixed effects, year fixed effects, and state specific time trends. Standard errors were clustered by state. Significant (p<0.05) estimates and 95% CIs are bolded.
