# Supplementary material for: State-level prescription drug monitoring program mandates and adolescent injection drug use in the United States, 1995–2017: A difference-in-differences analysis
Source: PLoS Med. 2020 Sep 25;17(9):e1003272. doi: 10.1371/journal.pmed.1003272 (PMC7518580; doi:10.1371/journal.pmed.1003272)
Supplement: S6 Table — (DOCX) [file pmed.1003272.s008.docx]

| **Variables** | **Reported Lifetime Injection Drug Use (N=331,025)** | |
| --- | --- | --- |
|  | Percentage Points | 95% CI |
| Lag: PDMP Mandate Implemented | **-1.48** | **-2.38 – -0.59** |
| PDMP (non-mandated) | 0.41 | -0.45 – 1.26 |
| Pill Mill law | 0.43 | -0.31 – 1.16 |
| Sex |  |  |
| Female | *Reference* |  |
| Male | **2.03** | **1.79 – 2.27** |
| Race/Ethnicity |  |  |
| White | *Reference* |  |
| Black/African American | **0.65** | **0.21 – 1.10** |
| Hispanic/Latinx | **1.59** | **0.30 – 2.20** |
| Other race/ethnicity | **2.05** | **1.25 – 2.85** |
| Age |  |  |
| 17 years of age | *Reference* |  |
| 18 years or older | **0.88** | **0.54 – 1.22** |
| Poverty | 0.02 | -0.11 – 0.15 |

**S6 Table.** Robustness Check Linear Difference-in-Differences "Lag" Analysis of PDMP Mandates in the 1-2 Years After: Adolescent Injection Drug Use in PDMP Mandate States Relative to Non-PDMP Mandate States

Note: Linear probability models include controls for state fixed effects, year fixed effects, and state specific time trends. Standard errors were clustered by state. Significant (p<0.05) estimates and 95% CIs are bolded.
