# Supplementary material for: State-level prescription drug monitoring program mandates and adolescent injection drug use in the United States, 1995–2017: A difference-in-differences analysis
Source: PLoS Med. 2020 Sep 25;17(9):e1003272. doi: 10.1371/journal.pmed.1003272 (PMC7518580; doi:10.1371/journal.pmed.1003272)
Supplement: S7 Table — (DOCX) [file pmed.1003272.s009.docx]

| **Variables** | **Reported Lifetime Injection Drug Use (N=227,968)** | |
| --- | --- | --- |
|  | Percentage Point | 95% CI |
| PDMP Mandate Implemented | **-1.46** | **-2.19 – -0.74** |
| Pill Mill law | 0.77 | -0.04 – 1.58 |
| Sex |  |  |
| Female | *Reference* |  |
| Male | **2.07** | **1.73 – 2.41** |
| Race/Ethnicity |  |  |
| White | *Reference* |  |
| Black/African American | **1.14** | **0.57 – 1.70** |
| Hispanic/Latinx | **1.56** | **0.86 – 2.26** |
| Other race/ethnicity | **1.59** | **0.83 – 2.35** |
| Age |  |  |
| 17 years of age | *Reference* |  |
| 18 years or older | **0.87** | **0.42 – 1.33** |
| Poverty | 0.07 | -0.08 – 0.24 |

**S7 Table.** Linear Difference-in-Differences Analysis of PDMP Mandates After non-Mandated PDMP Implementation: Adolescent Injection Drug Use in PDMP Mandate States Relative to Non-PDMP Mandate States

Note: Linear probability models include controls for state fixed effects, year fixed effects, and state specific time trends. Standard errors were clustered by state. Significant (p<0.05) estimates and 95% CIs are bolded.
